# Potential of biosurfactant as green pharmaceutical excipients for coating of microneedles: A mini review

**DOI:** 10.3934/microbiol.2024028

**Published:** 2024-07-30

**Authors:** Marzieh Sajadi Bami, Payam Khazaeli, Shayan Fakhraei Lahiji, Gholamreza Dehghannoudeh, Ibrahim M. Banat, Mandana Ohadi

**Affiliations:** 1 Pharmaceutics Research Center, Institute of Neuropharmacology, Kerman University of Medical Sciences, Kerman, Iran; 2 Department of Bioengineering, Biopharmaceutical Research Laboratory, Hanyang University, Seoul, South Korea; 3 School of Biomedical Sciences, Faculty of Life & Health Sciences, Ulster University, Coleraine BT52 1SA, Northern Ireland, UK

**Keywords:** biosurfactants, green pharmaceutical, excipients, microneedles, coating method

## Abstract

Microneedles, a novel transdermal delivery system, were designed to improve drug delivery and address the challenges typically encountered with traditional injection practices. Discovering new and safe excipients for microneedle coating to replace existing chemical surfactants is advantageous to minimize their side effect on viable tissues. However, some side effects have also been observed for this application. The vast majority of studies suggest that using synthetic surfactants in microneedle formulations may result in skin irritation among other adverse effects. Hence, increasing knowledge about these components and their potential impacts on skin paves the way for finding preventive strategies to improve their application safety and potential efficacy. Biosurfactants, which are naturally produced surface active microbial products, are proposed as an alternative to synthetic surfactants with reduced side effects. The current review sheds light on potential and regulatory aspects of biosurfactants as safe excipients in the coating of microneedles.

## 1. Introduction

**Figure 1.**
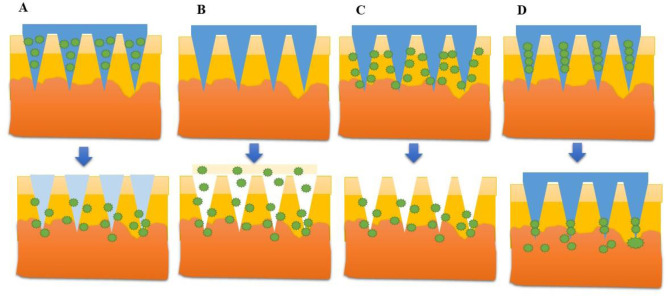
Schematic illustration of different kinds of microneedles: (A) Dissolving microneedles are constructed from biodegradable polymers; (B) Solid microneedles are used for pre-treatment of the skin and employ a poke with patch approach; (C) Coated microneedles use a coat and poke approach, where a drug solution is applied on the needle surface; (D) Hollow microneedles are filled with the drug solution and deliver the drug into the dermis.

Microneedles are sharp structures that measure less than a millimeter in length. These needles are used to inject drugs or vaccines by piercing the epidermis, which is the main barrier to transdermal drug delivery [1]. Microneedles overcome the limitations of hypodermic needles and reduce tissue damage and pain during application. In recent years, dissolving, solid, coated, and hollow microneedles have been developed and used as invasive drug delivery tools (Figure 1). Hollow microneedles are similar to hypodermic needles but are much shorter and are used to infuse liquid drug formulations through tiny bores. Solid microneedles are used to make small holes in the skin, followed by the application of a patch, while polymer microneedles are made from polymers that can dissolve [2]. Coated microneedles contain a layer of drug formulation coating over the solid microneedles, which dissolves upon application into the skin [3]. A wide range of ingredients can be transferred by coated microneedles, such as small molecules, proteins, DNA, viruses, and some microparticles [4]. The physical attachment of the microneedle surface and the drug is necessary for coating microneedles. This physical contact technique determines the coating method which vary including dip, inkjet, drop, spray, and immersioncoating [5]. Each coating method requires a different coating solution with a specific viscosity and surface tension. Various excipients can be administered to optimize the coating characteristics of the microneedles [6]. The most widely employed excipients used in coated microneedles are surfactants.

Surface-active agents, also known as amphiphiles or surfactants, tend to preferentially accumulate at the boundary or interface between two phases and reduce the surface tension [5],[7]. These agents are employed in a variety of applications, such as increased solubility, permeability, dissolution rate, and colloid stability. Nevertheless, most chemical surfactants are known to cause toxicity, resulting in global alerts and different regulations. Meanwhile, there are concernes regarding their biodegradability, safety, and eco-friendliness [8],[9]. In addition, crossing biological barriers leads to the direct contact of microneedles with viable tissue potentially causing disruptions or adverse reactions. Hence, the microneedle coating excipients such as surfactants ought to be an innocuous or biocompatible compounds [10]. Biocompatible coating excipients do not produce a toxic or immunological response following contact with tissues or bodily fluids [11]. Efforts are therefore focused on finding greener alternative biological surfactants that are less antagonistic, which are not only safer for humans and the environment but also are acceptable from an economic perspective [12]. Biosurfactants are typically extracellular surface active molecules produced by microbial culture and characterized by low toxicity and have a wide range of potential applications [13],[14]. This review proposes substituting synthetic surfactants with biosurfactants that have similar properties but fewer adverse effects as coating excipients.

## 2. Natural versus synthetic surfactants as excipients in microneedle coating solution

Chemical surfactants have been used for more than ten decades. Although chemical surfactants are a large and diverse family, they have a common attribute (i.e., the ability to reduce the surface tension between two immiscible phases, mostly water and oil) [15]. A typical surfactant has a lipophilic group, often a long-chain hydrocarbon moiety, and a hydrophilic group, which is the charged/uncharged polar group; hence, based on the charge status of their head groups, surfactants can be classified into cationic (e.g., quaternary ammonium compounds), anionic (e.g., soap, detergents, sodium dodecyl sulfate), non-ionic (e.g., poloxamer, Tween 80, Triton-X), and amphoteric surfactants (e.g., betaine) [16]. Because surfactant monomers are added into the solution, the surface tension will decrease until reaching the critical micelle concentration (CmC), which is the lowest concentration necessary to initiate the formation of micelles [17].

Due to the solution behavior of surfactants rooted in their particular molecular features, different surfactants are used as excipients in the microneedle coating solution [18]. The applied surfactants used in microneedle coatings include polymers such as Lutrol F68 NF/Poloxamer 188 (0.5% to 2% (w/v)) and Tween class of molecules (1% to 0.2% (w/v)) [3]. Surfactants are used as surface modification agents to help attenuate the surface tension of the coating solution, which translates into appropriate solubility of the drug particles on the surface of the microneedles [16]. Apart from increased stability or solubility of the drug, surfactants also act as wetting agents. Due to their wetting effect, surfactants are administered in formulations of coating solution to increase both the wetting and dispersion of drug particles that translates into maximizing the surface area of particles in the dissolution [19].

In addition, these agents have also been administered as emulsifying agents to increase penetration (in monomeric levels or at concentrations higher than the CmC) in topical formulations. Although chemical surfactants have clear advantages, they also possess two major disadvantages: unsustainability and problems related to biodegradability [20]. There are complexities regarding the application of surfactants in pharmaceutical products, which may even be beyond their intended purpose [21],[22]. Some studies have investigated the toxicological effects of surfactants [20]. Different classes of surfactants lead to various toxic cytological effects, including changing the permeability of the membrane, affecting its integrity, skin damage and irritation, and deactivation of enzymes [23]. The toxicity levels of synthetic surfactants depend on their structure and concentration [24]. Previous studies showed that non-ionic surfactants have lower toxicity while having the advantage of higher power solubility under biological conditions [25],[26]. While synthetic surfactants continue to be attractive excipients for coating solutions of microneedles, there is an understandable increasing interest in finding green and bio replacements for surfactants.

Biosurfactants contain several amphipathic molecules, each with its particular chemical structure, and are naturally produced by certain microorganisms [27],[28]. Nevertheless, while chemically synthesized surfactants have been categorized based on the status of their head group, the classification of microbial surfactants varies according to their chemical composition, microbial origin, and molecular weight. This classification system has resulted in two groups of low (e.g., lipopeptides and glycolipids) and high (e.g., lipoproteins, proteins, and polysaccharides) molecular weight surfactants [29]. The best-studied microbial surfactants are glycolipids and lipopeptides. Rhamnolipids produced by *Pseudomonas aeruginosa* and sophorolipids produced by *Candida bombicola* (now known as *Starmerella bombicola*) that contain monosaccharides or disaccharides, combined with long-chain aliphatic acids or hydroxyaliphatic acids are examples of glycolipids. Examples of lipopeptides include Surfactin, Iturin, and Fengicyn cyclic lipopeptides typically produced by *Bacillus* species as antibiotic molecules [29]. The CmC of efficient surfactants is low, which means lower levels of surfactants are required to reduce the surface tension to a particular level.

Biosurfactants are most efficient at their CmC, which may be lower than that of chemical surfactants by 10–40 times; hence, less surfactant is required to achieve maximum reduction in surface tension [30]. The other main feature of biosurfactants is their stability and unchanged activity following exposure to extreme environmental conditions [31]. Additionally, these bio-based amphiphilic molecules are easily degraded and have low or no toxicity [32]. Due to their biological and physicochemical characteristics, different types of biosurfactants have been used in the pharmaceutical industry [33]–[35]. As safe excipients, they appear as bioemulsifier, wetting, solubilizing, and dispersing agents in the drug delivery systems to enhance the bioavailability of some drugs with low aqueous solubility.

One of the fundamental properties of biosurfactants is their ability to reduce surface and interfacial tension. Interfacial tension could be reduced more effectively by biosurfactants than by chemical surfactants. Under harsh chemical and physical conditions, surfactin, an extremely active surface agent, displays 72 mN/m to 27 ± 2 mN/m expressive surface activity and 3.79 ± 0.27 mN/m to 0.32 ± 0.02 mN/m interfacial tension [36]. Biosurfactant chemical structure is considerabley more complex, which results in its characteristic surface properties. Biosurfectants differ from synthetic surfactants in their unclear polarity distribution and their branched or ring structures [37]. One quality that makes biosurfactants green excipients is their ability to dissolve hydrophobic compounds in aqueous solutions [38]. As solubilizing agents, biosurfactants function more effectively than synthetic surfactants. For instance, *Rhodococcus erythropolis* HX-2 yields a biosurfactant with higher solubilization than the synthetic surfactants Triton X-100, polysorbate 80 (Tween 80), and sodium dodecyl sulfate (SDS) [39]. Moreover, microbial surfactants could be proposed as alternative skin permeation enhancers to chemical surfactants [19]. Gupta et al reported a *Bacillus licheniformis* biosurfactant ointment formulation acted as a transdermal substitute for faster healing of impaired skin wounds [40].

Recently, two different biosurfactants isolated from human *Lactobacillus* strains were evaluated for possible application as transdermal permeation enhancers. Particularly, produced biosurfactants have showen to have a dual positive impact on hydrocortisone, which has been applied as model drug [19]. Proteins, lipids, and sugars, the elements of biosurfactants, resemble those the skin cell membrane is composed of (proteins and phospholipids), which is not the case for chemical surfactants. Furthermore, surface activity and lipophilicity determine the particle transfer across the skin membrane; hence, the biosurfactants' structure makes the skin cell membrane highly permeabile to them [41]. Also, *in vitro* evidence showed that surfactin, sophorolipids, and rhamnolipids are well suited for the human skin applications [42]. Several researchers reported the higher toxicity of chemical surfectants to mammalian cells compared to biosurfactants, making them much safer for use [43]. Apart from biosurfactants' administration as safe excipients to increase the physical and chemical properties of the pharmaceutical formulations, they can be administered to enhance the efficacy or bio performance of drugs, such as enhancing antimicrobial and antibiofilm activities of some drugs [35]. Major classes of biosurfactants and their applications are reported in Table 1.

**Table 1.** Major classes of biosurfactants and their application in pharmaceutical application.

Biosurfactant class	Example	Microorganism	Activity/potential application	Reference
Glycolipids	Rhamnolipids	*P. aeruginosa*	Bioemulsifier	[44]
	Sophorolipids	*C. bombicola*, *Candida apicola*	Bioemulsifier, antimicrobial agent	[45],[46]
	Rhamnolipids	*Rhodococcus fascians*	Solubilization activity	[47]
	Sophorolipids	*Starmerella bombicola*	Antimicrobial activity	[48]
Lipopeptides	Surfactin	*Arthrobacter* sp.	Surface activity agent	[36]
	Arthrofactin	*Arthrobacter* sp.	Surface activity agent	[49]
	Lipopeptide	*Lactobacillus. crispatus* BC1	Emulsification, permeation enhancer	[19],[50]
	Lipopeptide	*Lactobacillus. gasseri* BC9	Emulsification, permeation enhancer	[19],[50]
	Surfactin	*Bacillus subtilis* KLP2015	Antimicrobial and antitumor activity	[51]

## 3. Green surfactants as safe excipients for coating microneedles

**Figure 2.**
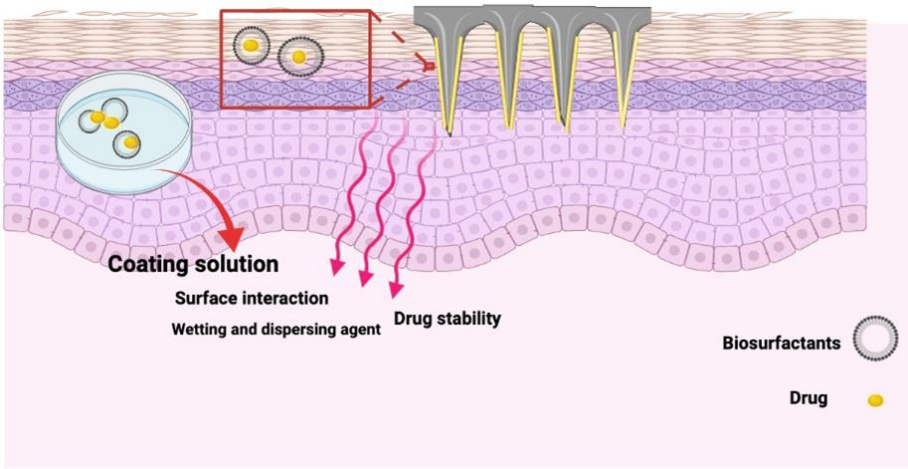
Schematic diagram showing biosurfactants facilitate delivery of the drug into the skin using coated microneedles as green excipients. Figure created using BioRender (https://biorender.com/).

It is widely known that the successful coating of microneedles (both *in vitro* and *in vivo*) can be significantly influenced by compounds such as coating excipients, namely surfactants [52]. Although there is evidence regarding their ability to increase permeation under certain circumstances, several studies have reported that topical use of synthetic surfactants in cosmetic or pharmaceutical industries may result in some problems for the users and side effects, such as contact dermatitis and irritation [19]. Hence, using new surface-active agents, namely biosurfactants, which do not lead to such issues, is currently quite appealing as a potential method for drug delivery [53]. As mentioned above, biosurfactants are comparable to their chemical counterparts and have higher biodegradability, lower toxicity, and lower CmC. In addition, their performance can be better than chemical surfactants in terms of surface activity and stability [53]. Here, we postulate that biosurfactants can be suitable excipients for coating microneedles and are suitable elements for increasing the wetting, dispersing, and reducing the surface tension of the coating solution (Figure 2).

## 4. What are the essential criteria of excipients coated on a microneedle array

Several criteria should be considered when selecting an excipient for coating microneedles, including but not limited to the biocompatibility of materials. Moreover, formulations approved by local regulatory agencies, such as the United States Food and Drug Administration (FDA) are preferable [54]. The FDA has published a guideline for non-clinical research intended to evaluate new pharmaceutical excipients, which emphasizes conducting all the necessary toxicology evaluations for potential new excipients following the state-of-the-art protocols and good laboratory practice regulations. In addition, the amount of the applied coating excipient should be as small as possible since applying larger amounts automatically results in the reduction of the active component in the coatings and translates into the reduced drug-carrying capacity of the microneedle array [53],[55].

## 5. Regulatory aspects of the hypothesis

### 5.1. Challenges, selection guidelines, and future prospects of biosurfactants in microneedle arrays

Several aspects should be studied to determine the potential of biosurfactants as safe excipients. The first aspect is evaluating the safety profile for the intended use and its administration route. Based on the regulations for pharmaceutical development, new biosurfactants developed for drug products should fulfill the criteria of efficacy, safety, stability, pharmacokinetics profile, and biological agreement with the other elements of the delivery system (i.e., drug and excipients) to ensure their safe administration by clinical routes [54]. As the production of biosurfactants is based on microbial culture, apart from the guidelines introduced to assess their toxicity profile, a series of impurity tests should be carried out before presenting them as new excipients [53]. The second criterion is about identifying the biosurfactant and its optimal administration dose [56]. For all surfactants, the CmC is the ideal point to start.

Another criterion is to investigate purified congeners of the biosurfactants wherever possible as most biosurfactant formulations are a collections of mixed congeners presenting an overall characteristic rather than an idividule conger properties which might be significantly different to each other [57]. Finally, the quality of green biosurfactants should be enhanced by using cost-effective methods, introducing new scale-up bioprocessing technologies, and developing novel characterization methods [58]. As large-scale industrial production can considerably hinder the administration of biosurfactants, several methods are introduced to enhance the economical production of these compounds, including processes intended to optimize the media components and growth conditions and the design of experiments for statistical optimization of media components [56]. Few studies have investigated the safety-related aspects of the administration of biosurfactants as excipients [53]. However, the efficacy of administering biosurfactants in cosmetic and antibiotic formulations has been established [59]. In addition, these compounds could fulfill the requirements announced by the related regulatory agencies for biocompatible and nontoxic excipients worldwide; hence, the way is paved for their successful implementation in drug delivery formulations.

## 6. Conclusions

Microneedles are one of the novel skin drug delivery systems that has solved weveral problems such as pain in the injection area, risk of infection, nonacceptance of the patient and nonpassage of the drug through the stratum corneum layer. However, the use of chemical compounds such as synthetic surfactants often leads to some side effects in the use of microneedles. Biosurfactants are considered as a natural alternative to synthetic surfactants. If biosurfactants can prove their safety in more studies according to FDA regulations and their production is optimized on a high scale, they can be used as a suitable alternative to reduce the side effects of chemical compounds used in microneedle. According to the approach of using safe and green compounds, more studies are necessary and expected in this field.

## Use of AI tools declaration

The authors confirm that they did not utilize any Artificial Intelligence (AI) tools in producing this article.
